# Baricitinib Ameliorates Experimental Autoimmune Encephalomyelitis by Modulating the Janus Kinase/Signal Transducer and Activator of Transcription Signaling Pathway

**DOI:** 10.3389/fimmu.2021.650708

**Published:** 2021-04-13

**Authors:** Chun Dang, Yaoheng Lu, Xingyu Chen, Qian Li

**Affiliations:** ^1^ West China Medical Publishers, West China Hospital, Sichuan University, Chengdu, China; ^2^ Department of General Surgery, Chengdu Integrated Traditional Chinese Medicine & Western Medicine Hospital, Chengdu, China; ^3^ Department of General Surgery, Chengdu University of Traditional Chinese Medicine Affiliated Traditional Chinese Medicine & Western Hospital, Chengdu, China; ^4^ Department of Neurology, The Second Affiliated Hospital of Harbin Medical University, Harbin, China

**Keywords:** baricitinib, EAE, JAK/STAT, Th1/Th17, Th1, Th17

## Abstract

Experimental autoimmune encephalomyelitis (EAE) is an animal model of multiple sclerosis (MS) and a CD4+ T cell-mediated autoimmune disease. The Janus kinase (JAK)/signal transducer and activator of transcription (STAT) pathway is recognized as the major mechanism that regulates the differentiation and function of T helper (Th) 1 and Th17 cells, which are recognized as pivotal effector cells responsible for the development of EAE. We used baricitinib, a JAK 1/2 inhibitor, to investigate the therapeutic efficacy of inhibiting the JAK/STAT pathway in EAE mice. Our results showed that baricitinib significantly delayed the onset time, decreased the severity of clinical symptoms, shortened the duration of EAE, and alleviated demyelination and immune cell infiltration in the spinal cord. In addition, baricitinib treatment downregulated the proportion of interferon-γ+CD4+ Th1 and interleukin-17+CD4+ Th17 cells, decreased the levels of retinoic acid-related orphan receptor γ t and T-bet mRNA, inhibited lymphocyte proliferation, and decreased the expression of proinflammatory cytokines and chemokines in the spleen of mice with EAE. Furthermore, our results showed the role of baricitinib in suppressing the phosphorylation of STATs 1, 3, and 4 in the spleen of EAE mice. Therefore, our study demonstrates that baricitinib could potentially alleviate inflammation in mice with EAE and may be a promising candidate for treating MS.

## Introduction

Multiple sclerosis (MS) is an autoimmune-mediated disease of the central nervous system (CNS) and is characterized by inflammation and neurodegeneration (1). MS is among the most common causes of neurological disability in young adults worldwide (2). In approximately 85% of these patients, the disease starts with neurologic dysfunction, followed by periods of remission, relapse, and/or a progressive disease course (3). Although the specific details of MS pathogenesis are not clear, CD4+ T cell-mediated autoimmunity is considered the most critical component (4). Both the development of CD4+ T cells into various T helper (Th) cell subtypes and the production of cytokines are essential for the pathogenesis of MS (5). Experimental autoimmune encephalomyelitis (EAE) is a T cell-driven autoimmune disease of the CNS that shares strong similarities with MS in terms of clinical and histopathological features (6).

CD4+ T cells are categorized into four major subsets according to cytokine secretion and transcription factor expression: T helper type 1 (Th1), Th2, Th17, and regulatory T (Treg) cells (7). Retinoic acid-related orphan receptor γ (RORγt) is very important for the differentiation and proliferation of Th17 cells. T-bet, GATA-3, and Foxp3 are of critical importance to Th1, Th2, Treg, and Th17 cells, respectively (8). The pathogenesis of MS and EAE is associated with numerous cytokines. The signature cytokines interferon γ (IFNγ) and interleukin (IL)-23 partly promote the differentiation of Th1 and Th17 cells (9, 10). Th1 cells are closely involved in mediating the pathology of EAE. Th17 cells have been identified as pivotal cells in autoimmune inflammatory demyelination in EAE rodent models (11). The inhibition of Th17 cells leads to the amelioration of EAE, whereas the adoptive transfer of Th17 cells directly increases the severity of EAE (12, 13).

The Janus kinase (JAK)/signal transducer and activator of transcription (STAT) pathway is crucial for initiating innate immunity and ultimately constraining immune responses in immune-mediated diseases (14). In MS and EAE, there is much evidence for the aberrant functionality of the JAK/STAT pathway (15). The JAK/STAT pathway is recognized as the primary mechanism regulating the differentiation and function of Th1 and Th17 cells, which are recognized as pivotal effector cells responsible for the development of EAE (16). IFN-γ and IL-12 bind to the receptors of naive CD4+ T cells, which drive Th1 differentiation by activating their downstream transcription factors (STAT1 and STAT4) (17). IL-6 activates and stimulates tyrosine phosphorylation of STAT3, a critical transcription factor for the induction of pathogenic Th17 cells (18). The loss of STAT3 in T cells prevents the development of EAE (19).

Baricitinib is a novel immunosuppressant approved for use in Europe and Japan to treat adults with rheumatoid arthritis (RA) (20). It blocks the action of JAK 1, 2 as a selective inhibitor of JAK1 and JAK2 (21, 22). Baricitinib has been shown to have beneficial effects on suppressing the downstream activation of STATs, particularly STAT3, and inhibits the intracellular signaling of multiple proinflammatory cytokines, including IL-6 and IL-23, in rodent models of RA (23). However, the role of baricitinib in EAE remains unclear. In this study, we investigated the protective properties of baricitinib and the efficacy of inhibiting the JAK/STAT pathway in a rodent model of EAE.

## Material and Methods

### Animals

Female wild-type C57BL/6J mice were purchased from Vital River Corporation Company, Ltd. (Beijing, China). Mice were used when they were 6 to 8 weeks old. All experimental mice were maintained under specific pathogen-free conditions. All animal experiments were designed and performed in accordance with the guidelines of Animal Research: Reporting of *In Vivo* Experiments (24). The experiments were conducted in a blinded manner to avoid bias, and the mice were randomly assigned to different experimental conditions. All experiments were approved by the Animal Experiments Ethical Committee of Chengdu University of Traditional Chinese Medicine.

### Induction of EAE and Measurement of the Neurologic Symptoms

Mice were injected subcutaneously with 200 μg of myelin oligodendrocyte glycoprotein (MOG)35-55 peptide (GenScript, Nanjing, China). The injection was prepared by mixing MOG35-55 in complete Freund’s adjuvant (Difco, Detroit, MI, USA) containing 500 μg of nonviable desiccated *Mycobacterium tuberculosis* (Difco) into the hind flank. Mice received 200 ng of pertussis toxin (List Biological, Campbell, CA, USA) intraperitoneally on the day of the injection and 2 d later. The symptoms were assessed as follows: 0, no clinical signs; 1, decreased tail tone; 2, partial paralysis; 3, paraplegia; 4, full paralysis; and 5, moribund state. An EAE evaluation and clinical score assessment was performed daily, as described previously, from the day of the injection until day 30 (25).

### Study Design and Drug Administration

Baricitinib (INCB028050, Selleck, Shanghai, China) was suspended in 0.5% methylcellulose (Sigma, St. Louis, MO, USA) and administered by oral gavage (23). The mice were randomly assigned to three groups according to the treatment they received: the vehicle group, baricitinib-1 group (3 mL/kg) and baricitinib-2 group (10 mL/kg) as described previously (20, 23). The concentration of baricitinib was 1 mg/mL in the baricitinib-1 and baricitinib-2 groups. Oral doses differed between the two experimental groups. Baricitinib was administered from day 0 to 14 after the injection for the treatment protocol. Under similar experimental conditions, the control groups were treated with vehicle - 0.5% methylcellulose.

### Histology

For histological analysis, the spinal cord was harvested at the peak of EAE. The tissue was prepared for paraffin embedding and sectioning (8 μm thick). The sections were deparaffinized, rehydrated, and stained with Luxol Fast Blue (LFB) following standard immune cell infiltration or demyelination analysis procedures. Staining was evaluated using a Nikon digital light microscope (Tokyo, Japan).

### Real-Time Quantitative Polymerase Chain Reaction (PCR)

The mice were sacrificed at the peak of EAE after baricitinib treatment, and splenic cells were harvested after lysing the red blood cells. Total RNA was extracted from splenic mononuclear cells (MNCs) lysed in TRIzol reagent (Invitrogen, Carlsbad, CA, USA) and reverse transcribed into cDNA using TransScript First-Strand cDNA Synthesis SuperMix (TransGen Biotech, Beijing, China). Quantitative real-time PCR was performed according to published methods on a Bio-Rad PCR Detection System (Bio-Rad, Hercules, CA, USA (26). The following mice-specific primers were used to measure gene expression: RORγt (sense, 5′-GGTCCAGACAGCACTGCATTC-3′; antisense, 5′-GGTGCGCTGCCGTAGAAGGT-3′), t-bet (sense, 5′-CAGTTCAACAGCACCAGAC AG-3′; antisense, 5′-CCACCAAGACCACATCCACAAA-3′), GATA-3 (sense, 5′-GAAGGCATCCAGACCCGAAAC-3′; antisense, 5′-ACCCATGGCGGTGACCATGC-3′), and Foxp3 (sense, 5′-CTCTAGCAGTCCACTTCACCAA-3′; antisense, 5′-CACCCACCCTCAATACCTCTCT-3′). GAPDH (sense, 5′- TGTGATGGGTGTGAACCACGAGAA-3′; antisense, 5′-CATGAGCCCTTCCACAATGCCAAA-3′). The relative gene expression was normalized to that of the housekeeping gene glyceraldehyde 3-phosphate dehydrogenase (GAPDH) and calculated using the 2-ΔΔCt method.

### Cell Cultures, T Cell Isolation, and Sorting

Purified murine CD4+ T cells were isolated from the splenic cells using a MojoSort™ Mouse CD4 T cell isolation kit (BioLegend, San Diego, CA, USA) as described previously (27). The purity of the isolated cells was routinely 95%, as determined by flow cytometry analysis for downstream studies (28). The cells were cultured in RPMI 1640 medium (Gibco, Waltham, MA, USA) supplemented with 10% fetal bovine serum (v/v) (Gibco) and 1% penicillin-streptomycin solution (Solarbio, Beijing, China) (37°C, 5% CO_2_).

### Flow Cytometry

In the peak phase of EAE in mice, splenic MNCs and brain cells were harvested as described previously (29). For intracellular cytokine staining, splenic MNCs were restimulated in complete 1640 medium with a cell activation cocktail containing Brefeldin A (BioLegend) for 6 h. The cells were surface-stained with an anti-CD4 antibody for 30 min. After washing and fixation, the cells were stained with anti-IFN-γ (BioLegend) for Th1 cells, anti-IL-4 (BioLegend) antibody for Th2 cells, and anti-IL-17 (BioLegend) for Th17 cells. To quantify the Treg cells, the cells were surface-stained with anti-CD4 (BioLegend) and anti-CD25 (BioLegend) without the stimulation protocol. After fixation, permeabilization buffer was used as a washing solution (BioLegend), and the cells were then stained with anti-Foxp3 antibodies. The antibodies were tagged with phycoerythrin, allophycocyanin, or fluorescein isothiocyanate. Data were acquired on a FACSAria flow cytometer (BD Bioscience, San Jose, CA, USA) and analyzed using FlowJo software (Ashland, OR, USA).

### Western Blot Analysis

Splenic MNCs from vehicle-treated and baricitinib-treated EAE mice were restimulated with MOG35-55 peptide (10 μg/mL) for 24 h (30). Proteins from splenic cells were obtained using RIPA buffer containing protease inhibitors (Invitrogen). All procedures were performed for western blot analysis as described previously (26). The membranes were incubated at 4°C overnight with the following primary antibodies and dilutions: rabbit anti-phospho-JAK1 (1:1000; Cell Signaling Technology, Danvers, MA, USA), mouse anti-JAK1 (1:500; Cell Signaling Technology), rabbit anti-phospho-JAK2 (1:2000; Cell Signaling Technology), rabbit anti-JAK2 (1:500; Cell Signaling Technology), mouse anti-phospho-STAT1 (1:500; Cell Signaling Technology), mouse anti-STAT1 (1:1000; Cell Signaling Technology), rabbit anti-phospho-STAT3 (1:1000; Cell Signaling Technology), mouse anti-STAT3 (1:2000; Cell Signaling Technology), rabbit anti-phospho-STAT4 (1:2000; Cell Signaling Technology), rabbit anti-STAT4 (1:1000; Cell Signaling Technology), and mouse anti-β-actin (1:1000; Thermo Fisher Scientific),. Signals of specific protein bands were detected using a Gel Doc image analyzer (Bio-Rad, Hercules, CA, USA). The number of targeted proteins was normalized to that of β-actin. The number of targeted proteins was normalized to that of β-actin. β-Actin as the housekeeping protein was used to normalize the expression levels of JAKs, p‐JAKs, STATs, and p‐STATs. The ratios of p‐JAKs and p‐STATs to total JAKs and STATs were determined. The relative protein expression of active JAKs and STATs (phosphorylated/total) was analyzed (31–33).

### Cell Proliferation [3-(4,5-Dimethylthiazol-2-yl)-5-(3-carboxymethoxyphenyl)-2-(4-sulfophenyl)-2H-tetrazolium] (MTS) Assay

An MTS assay (Promega, Madison, WI, USA) was performed to investigate antigen-specific lymphocyte proliferation. At the peak of EAE, the spleens were removed under aseptic conditions, and splenic MNCs were harvested quickly. The cell samples were stimulated with 10 μg/mL of MOG35-55 peptide for 48 h. MTS (5 μg/mL) was then added to each well and incubated for 4 h. Absorbance was measured at 490 nm using a microplate reader (Thermo). The experiments were repeated in triplicate. The blank group contained fresh medium and MTS solution without splenic cells. The control group included fresh medium and the MTS solution with splenic cells. The experimental group included MOG35-55 peptide medium and MTS solution with splenic cells. Cell proliferation was determined according to the manufacturer’s protocol. The optical density (OD) values of the three replicate wells were averaged. The numerical value of the experimental group was calculated by subtracting the OD value of the blank group from the OD value of the experimental group. The numerical value of the control group was calculated by subtracting the OD value of the blank group from the OD value of the control group. The calculation result of the experimental group was divided by the calculation result of the control group, and finally multiplied by 100 (34).

### Enzyme-Linked Immunosorbent Assay (ELISA)

The mice were sacrificed at the peak of EAE from the three groups. Splenocytes were cultured with the MOG35-55 peptide at 10 mg/mL for 48 h. The supernatants of the splenic MNCs were collected. A simultaneous quantitative analysis of 12 cytokines, including IL2, IL4, IL5, IL6, IL10, IL12, IL13, IL17A, IL23, IFN-γ, TNFα, and TGFβ, was performed using a multi-analyte ELISA array kit (QIAGEN, Düsseldorf, Germany) according to the manufacturer’s instructions. The measurements were repeated in triplicate.

### Immunohistochemical Procedures

The spinal cord of EAE mice were harvested after anesthesia. The tissues were fixed with 4% paraformaldehyde and prepared for frozen sectioning by dehydrating with 15% sucrose solution, followed by 30% sucrose solution, and then embedding in OCT medium (Sakura, Torrance, CA, USA). Frozen sections (8 mm) were prepared with a freezing microtome (Thermo Fisher Scientific, Waltham, MA, USA) and mounted onto slides. Frozen sections were incubated with rabbit anti–myelin basic protein antibody (MBP; 1:500; Santa Cruz Biotechnology, Dallas, TX, USA), rabbit anti-Iba-1 (1:500, Abcam,Cambridge, MA, USA); rabbit anti-GFAP (1:1000, Abcam) overnight at 4°C. Frozen sections were incubated with Alexa Fluor 546–conjugated donkey anti-rabbit IgG (H+L) (1: 1000; Thermo Fisher Scientific) and Alexa Fluor 488–conjugated donkey anti-rabbit IgG (H+L) (1: 1000; Thermo Fisher Scientific), for 1 h at room temperature in dark. Sections were covered with DAPI (Abcam) to label all nuclei. Immunostaining slices was observed and pictured with a Nikon digital fluorescent microscope (Tokyo, Japan). The microglia and astrocytes counts were recorded and compared according to published method (35). Quantification of images was performed using Sigma Scan Pro 5.0 software (SPSS, Chicago, IL, USA). As this software measured the degree of brightness, the LFB images were firstly inverted so that the apparent amount of myelin corresponded to the degree of brightness showing in the image. Measurement setting was continuously kept in a tracing mode with the line width in pixels equaling to 1. The average intensity was successively measured in each the region of interest (ROI). To minimize inter-section variability due to a variable level of staining, the average ROI values were normalized to the average intensity within the white matter. The lateral white matter was chosen as no damage is expected in this area. Quantification of the MBP images was performed using the same procedure without the image inversion procedure (36).

### Statistical Analyses

All statistical analyses were performed using GraphPad Prism (version 7.0; San Diego, CA, USA). The Mann-Whitney U test was used to evaluate the differences between the two groups. One-way analysis of variance and the Kruskal-Wallis test followed by a Tukey *post hoc* test were used to analyze the differences among multiple groups. Statistical significance was set at p < 0.05. Data are presented as the mean ± SEM.

## Results

### Baricitinib Attenuates EAE Clinical Symptoms

**Figure 1:**
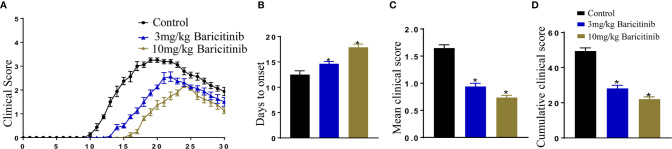
Baricitinib delays and attenuates the symptoms of experimental autoimmune encephalomyelitis (EAE) in mice. Mice were injected with MOG35-55 peptide to establish the EAE model and received an injection of either baricitinib or vehicle. Day 0 is EAE induction. **(A)** The clinical symptom scores of the mice in the different groups after EAE induction. In the baricitinib-treated groups, the clinical signs were remarkably mild. The clinical scores were remarkably better in the baricitinib-treated groups than in the control group from day 10 to 30. **(B)** Baricitinib delayed the onset of disease in the baricitinib-treated groups. **(C)** The mean score for mice in each group was recorded every day over a 30 d period. Baricitinib improved the mean clinical score in mice with EAE. **(D)** The mean cumulative clinical score of vehicle-treated mice was significantly greater than that of baricitinib-treated mice (sum of scores = 30 d). Quantitative data are the mean ± SEM. *p < 0.05; n = 8 per group. SEM, standard error of the mean.

To confirm whether baricitinib treatment can alleviate the clinical manifestations of EAE, EAE models were induced using an injection of MOG35-55 peptide, after which the mice were divided randomly into baricitinib-1, baricitinib-2, or the control groups. After the induction of EAE, the timing and severity of EAE differed between the baricitinib therapeutic and control groups. The peak scores were delayed in the baricitinib-1 and baricitinib-2 groups compared to those in the control group (**Figure 1A**). In the control group, the behavioral and neurological scores began to increase on day 10, whereas in the baricitinib-1 group, symptoms were delayed, and the scores of the baricitinib-1 group started to increase around day 13, and the scores of the baricitinib-2 group increased around day 15 (**Figure 1B**). There was a significant decrease in the mean clinical score in the therapeutic groups compared to that in the control group (**Figure 1C**). The mean cumulative clinical score of the control group was significantly higher than that of the baricitinib therapeutic group (**Figure 1D**). Therefore, baricitinib treatment alleviated the neurological severity and disease progression of EAE.

### Effects of Baricitinib Treatment on Histologic Changes and Immune Cell Infiltration of EAE

**Figure 2:**
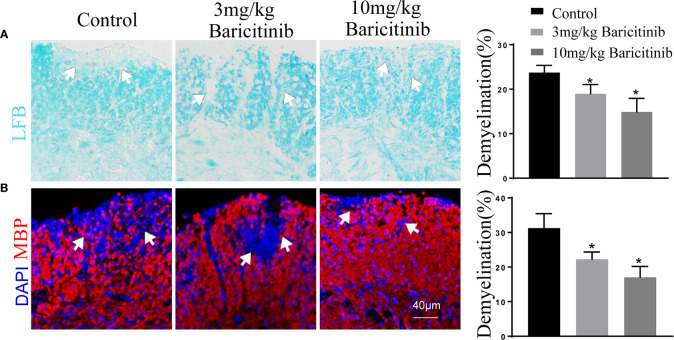
Baricitinib reduces pathological tissue injuries in experimental autoimmune encephalomyelitis (EAE) mice. The extent of demyelination was semi-quantitatively analyzed. The results from each group are displayed as representative images, and the statistics of infiltration and demyelination are shown. Demyelination of the central nervous system (CNS) was evaluated by Luxol Fast Blue (LFB) **(A)** and myelin basic protein (MBP) **(B)**. Cell nuclei were counterstained with DAPI (blue). Demyelination of the CNS was remarkedly reduced in the baricitinib-treated groups compared to that in the control group. The mean percentage of the microscopic fields with the lumbar spinal cord. Quantitative data are the mean ± SEM. *p < 0.05; n = 6 per group. SEM, standard error of the mean.

**Figure 3:**
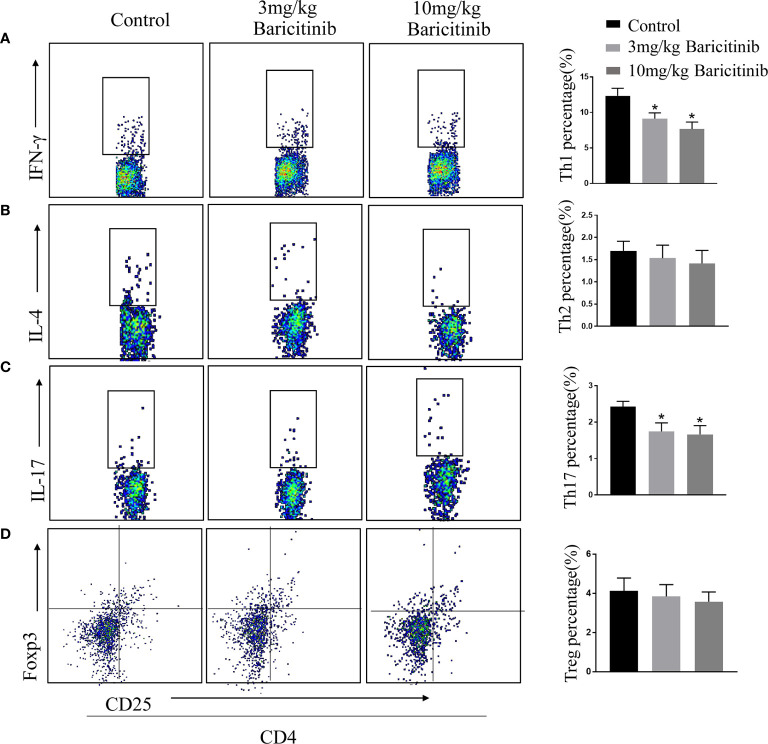
Baricitinib affects the differentiation of T-cells in CNS. At the peak phase of experimental autoimmune encephalomyelitis (EAE), CNS cells were isolated for flow cytometry to analyze changes in the differentiation of lymphocytes in spinal cord. The proportions of CD4+IFN-γ+Th1 **(A)**, CD4+IL-4+Th2 **(B)**, CD4+IL-17+ Th17 **(C)** and CD4+CD25+Foxp3+ Treg cells **(D)** of mice from different groups were investigated through flow cytometry analysis. The percentages of CD4+IFN-γ+ Th1 cells and CD4+IL-17+ Th17 cells in the CNS cells were decreased in both the baricitinib-treated groups than in the control group. Compared with the control group, baricitinib treatment did not affect the differentiation of CD4+IL-4+Th2 cells and CD4+CD25+Foxp3+ Treg cells. Quantitative data are the mean ± SEM. *p < 0.05; n = 6 per group.

**Figure 4:**
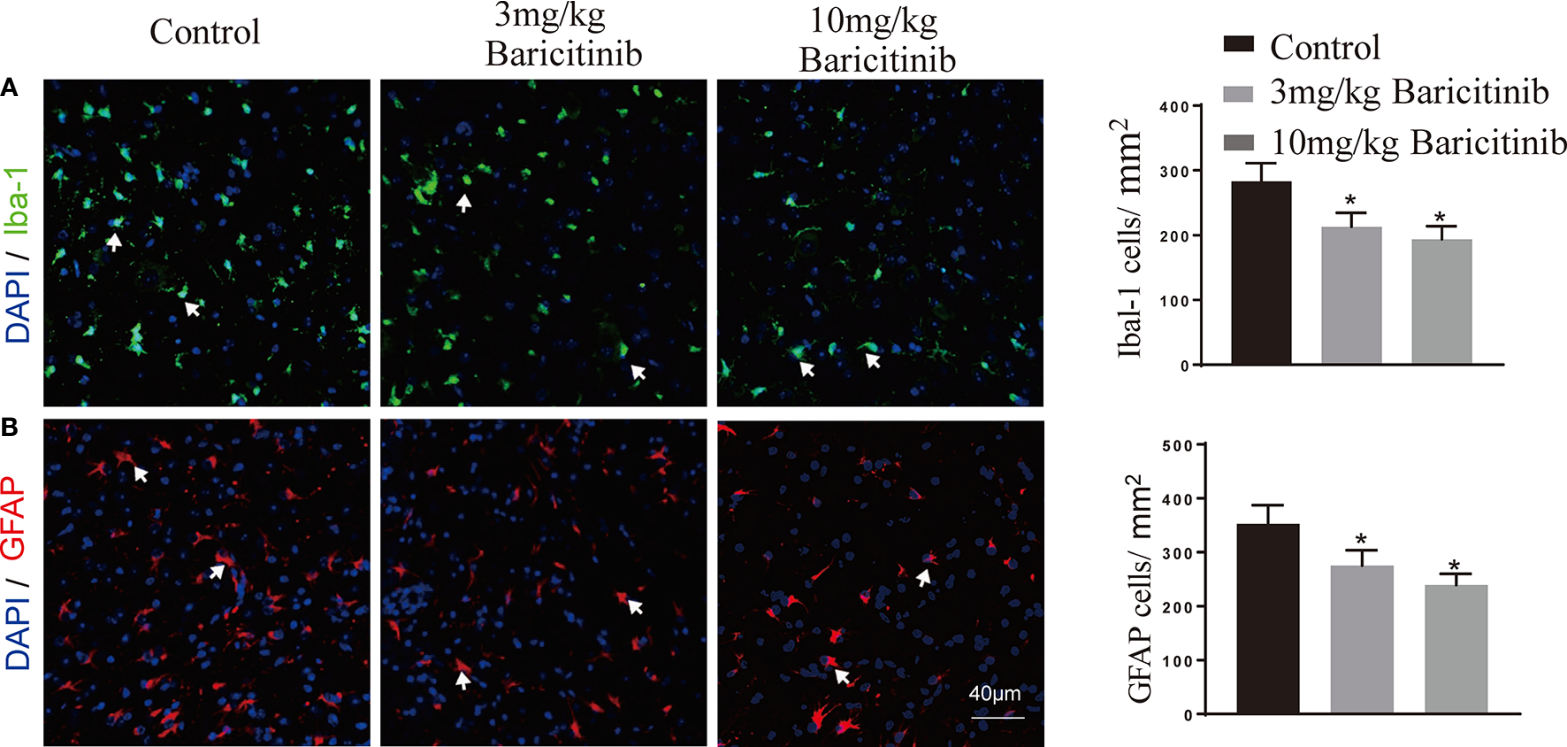
Representative images and distribution of microglia (Iba-1) and astrocytes (GFAP) in spinal cord of EAE mice. **(A)** Significantly lower Iba-1 positive cells (microglia) count was recorded in the baricitinib-treated groups when compared with the control group. **(B)** Representative immunostained images showing the distribution of astrocytes. GFAP positive cells (astrocytes) decreased significantly in the baricitinib-treated groups when compared with the control group. 5 sections per animal. Quantitative data are the mean ± SEM. *p < 0.05; n = 6 per group.

To determine whether the benefits of baricitinib on the neurological manifestation of EAE were related to the demyelination of lumbar spinal cords, the mice were sacrificed at the peak phase, and the lumbar spinal cords were removed for Luxol Fast Blue and myelin basic protein immunostaining. As shown in **Figures 2A** and **B**, baricitinib treatment significantly reduced the degree of demyelination (**Figures 2A, B**). Our data demonstrated that in the CNS, the baricitinib therapeutic group showed a significant decrease in the percentage of CD4+IFN-γ+ Th1 and CD4+IL-17+ Th17 cells (**Figures 3A, C** in both treatment groups). However, compared with the control group, the baricitinib treatment group did not differ significantly in the percentage of CD4+IL-4+ Th2 and CD4+CD25+Foxp3+ Treg cells (**Figures 3B, D**). The baricitinib-treated groups showed a lower count of microglia than the control group (**Figure 4A**). Furthermore, we evaluated the distribution of astrocytes and found a prominent decrease in the baricitinib-treated groups when compared with that in the control group (**Figure 4B**). Quantitation of immune cell infiltration and demyelination indicated that baricitinib treatment significantly reduced the clinical severity and CNS inflammation.

### Baricitinib Inhibits T Cell Proliferative Response

**Figure 5:**
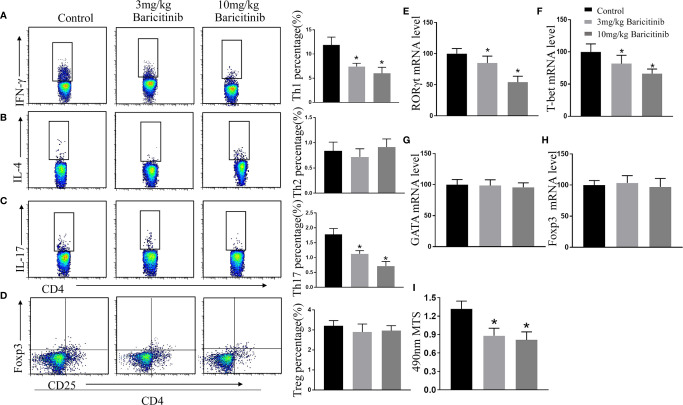
Baricitinib treatment has an immunoregulatory effect on the spleen of experimental autoimmune encephalomyelitis (EAE) mice. Splenic mononuclear cells (MNCs) were isolated for flow cytometry to analyze the percentage of CD4+IFN-γ+ Th1 **(A)**, CD4+IL-4+ Th2 **(B)**, CD4+IL-17+ Th17 **(C)**, and CD4+CD25+Foxp3+ Treg cells **(D)** at the peak phase of EAE. The percentages of Th1 and Th17 cells in the splenic MNCs were decreased in baricitinib-treated mice compared with those in vehicle-treated mice. However, baricitinib did not affect the differentiation of CD4+IL-4+ Th2 cells and CD4+CD25+Foxp3+ Treg cells. **(E–H)** Quantification of the mRNA expression of T-bet, GATA, RORγt, and Foxp3 in the baricitinib-treated groups and the control group. **(I)** MTS proliferation assays were performed to investigate the level of splenic lymphocyte proliferation. The results are displayed as the stimulation index. Baricitinib-treated groups exhibited significant reductions in proliferation. Quantitative data are the mean ± SEM. *p < 0.05; n = 6 per group. SEM, standard error of the mean.

CD4+ T cells play disease-promoting roles in the clinical progression of EAE in mice. Therefore, we evaluated the effect of baricitinib on the proportion of the different T cells. At the peak of EAE, we explored the influence of baricitinib on CD4+ T cell subpopulations in the spleen. Flow cytometric analysis revealed that baricitinib treatment markedly decreased the percentage of Th1 (CD4+ IFNγ+) and Th17 (CD4+ IL-17+) cells in CD4+ T cells compared to those in the control group (**Figures 5A, C**). Flow cytometry results showed that the percentages of Th2 (CD4+IL-4+) cells and Treg (CD4+CD25+Foxp3+) cells of spleen MNCs from the different groups were similar (**Figures 5B, D**).

T-bet, GATA, RORγt, and Foxp3 play essential roles in the homeostasis of Th1, Th2, Th17, and Treg cells. Therefore, the relative mRNA levels of T-bet, GATA, RORγt, and Foxp3 in the spleen were determined. Compared with the control treatment group, the baricitinib treatment effectively reduced the EAE-associated increase in mRNA levels of T-bet and RORγt (**Figures 5E, F**), and there were no significant differences in the relative mRNA levels of GATA and Foxp3 compared to those of the vehicle treatment (**Figures 5G, H**).

### Effects of Baricitinib on Lymphocyte Proliferative Responses

The effect of baricitinib treatment on lymphocyte proliferation was evaluated using the MTS assay. Lymphocyte proliferation was significantly reduced in the treatment groups compared to that in the control group (**Figure 5I**).

### Baricitinib Regulates CD4+ T Cell Differentiation Through Modulating the JAK/STAT Pathway

**Figure 6:**
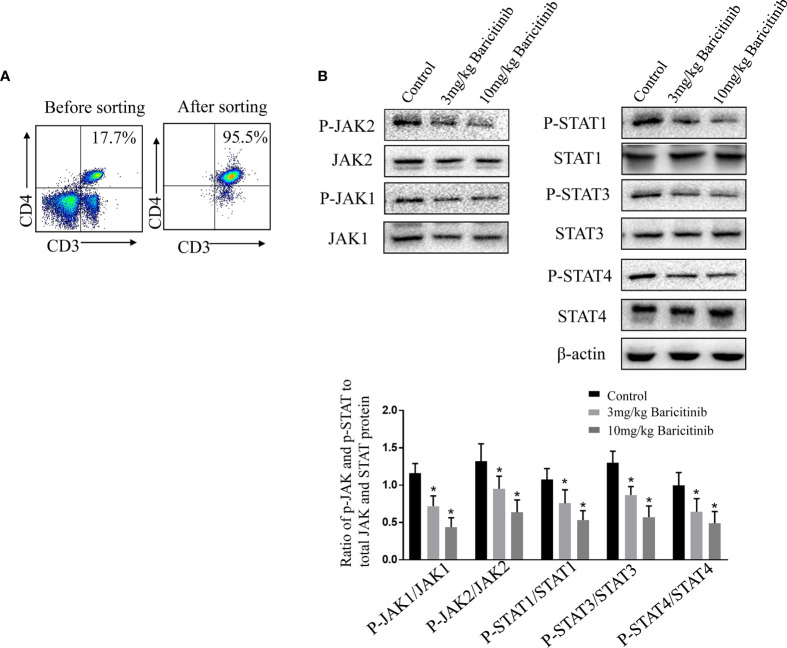
Baricitinib causes a CD4+ T cell phenotype shift by inhibiting the JAK/STAT pathway. **(A)** Representative flow cytometry plots show naive CD4+ T cells from the spleens were purified with magnetic microbeads, producing a purity of ≈95%. **(B)** Ratio of p‐JAK and p‐STAT to total JAK and STAT protein. Purified CD4+ T cells isolated from vehicle or baricitinib-treated EAE mice were restimulated with MOG 35-55 peptide (10 μg/mL). Western blot analysis of phosphorylated and total JAK1, JAK2, STAT1, STAT3, and STAT4 proteins were detected by immune-blotting. Total JAK1, JAK2, STAT1, STAT3, STAT4, and β-actin proteins as loading control were detected by immunoblotting. Quantitative data are the mean ± SEM. *p < 0.05; n = 6 per group. SEM, standard error of the mean.

To further investigate the mechanism underlying the beneficial effects of baricitinib in EAE, we assessed possible baricitinib-related changes in CD4+ T cells from the spleens of EAE mice. Naive CD4+ T cells were sorted and cultured with stimulation (MOG35-55 peptide) for western blot analysis (**Figure 6A**). The protein expression of several relevant transcriptional regulators was investigated in the treatment and control groups. STAT3 has been shown to serve as a master regulator of Th17 cell differentiation, and STAT1 and STAT4 activate Th1 differentiation. Western blot analyses of the spleen samples confirmed a lower level of pJAK1 and pJAK2 expression in the baricitinib-treated group than in the control group (**Figure 6B**). The baricitinib treatment group had a lower relative expression of pSTAT1, pSTAT3, and pSTAT4 in their spleens than the control group (**Figure 6B**).

### Effects of Baricitinib Treatment on Cytokine Profiles

**Figure 7:**
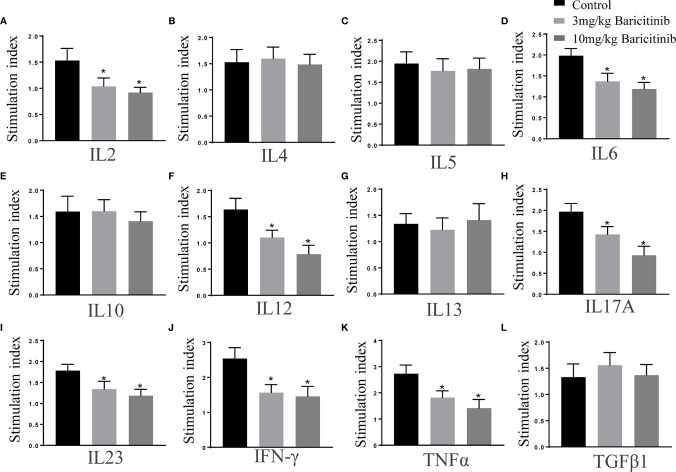
Baricitinib treatment alters the cytokine profiles. Splenic mononuclear cells (MNCs) were isolated on the peak day. Cytokine profiles of supernatants cultured with MOG 35-55 peptide (10 μg/mL) for 72 h were investigated with ELISA kits. **(A–L)** The levels of IL-2, IL-6, IL-12, IL-17, IL-23, IFN-γ, and TNF-α significantly reduced in the baricitinib treatment groups compared with those in the vehicle group. However, levels of IL-4, IL-5, IL-10, TGFβ, and IL-13 were not significantly different. Quantitative data are the mean ± SEM. *p < 0.05; n = 6 per group. SEM, standard error of the mean.

The effects of baricitinib on cytokine production were assessed simultaneously using multi-analyte ELISA array kits. As shown, in splenic MNCs stimulated with MOG35-55 peptide (10 μg/mL), the expression of cytokines IL-2, IL-6, IL-12, IL-17, IL-23, IFN-γ, and TNF-α were markedly decreased in the treatment groups compared to that in the control group (**Figure 7**). The expression levels of cytokines IL-4, IL-5, IL-10, TGFβ, and IL- 13 were not significantly different (**Figure 7**).

## Discussion

In this study, we provided evidence that the inhibition of the JAK/STAT pathway specifically inhibited STAT1, STAT3, and STAT4 activation and attenuated clinical disease in EAE models. Treatment with baricitinib, a potent inhibitor of JAK1 and JAK2, attenuated immune cell infiltration of the CNS, inhibited STAT activation, regulated differentiation and proliferation of CD4+ cells, and decreased the expression of proinflammatory cytokines and chemokines.

**Figure 8:**
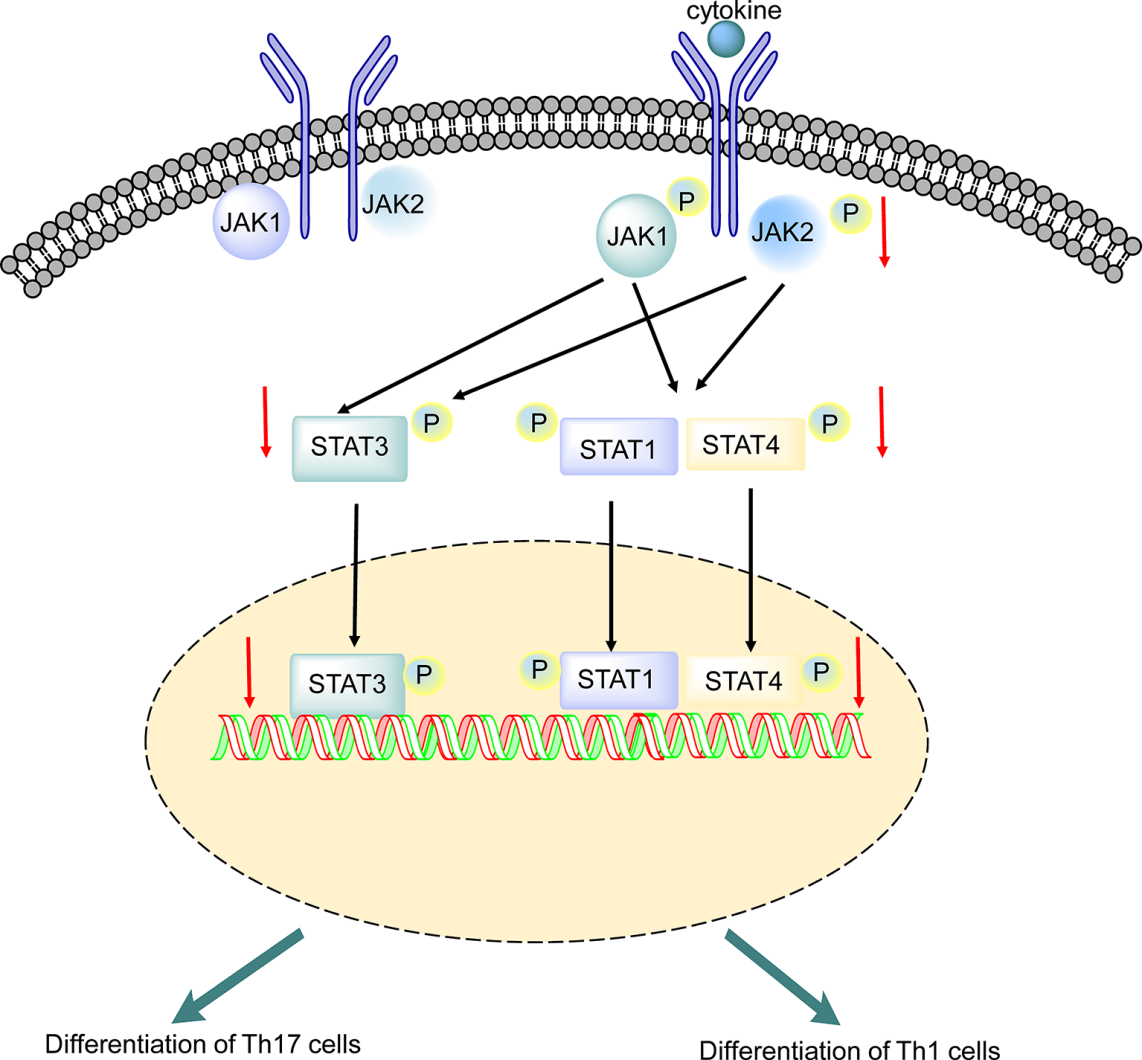
Putative mechanism underlying the effect of baricitinib on experimental autoimmune encephalomyelitis (EAE). Phosphorylation of receptor chains by JAKs is induced by cytokine binding. STAT1, STAT3, and STAT4 are phosphorylated by JAK1 and JAK2, leading to dimerization. Baricitinib inhibited JAK1 and JAK2, leading to decreased phosphorylation of STAT1, STAT3, and STAT4. The reduction in STAT3 phosphorylation may directly inhibit Th 17 cell differentiation. The reduction in STAT1 and STAT4 phosphorylation suppresses Th 1 cell differentiation. SEM, standard error of the mean.

Baricitinib, an adenosine triphosphate competitive kinase inhibitor, selectively inhibits JAK1 and JAK2 (37). Baricitinib is currently being studied in autoimmune diseases and has been approved for the treatment of RA in adults (23). In the JAK/STAT signaling pathway, STATs are phosphorylated and activated by JAKs (17). Baricitinib modulates the phosphorylation and activation of STATs by partially inhibiting JAK1 and JAK2 (38). This is the first study to report the efficacy of baricitinib in a rodent model of EAE. In this study, we investigated the effects of baricitinib on the self-reactive immune response in EAE mice *via* the JAK/STAT signaling pathway (**Figure 8**).

The pivotal role of pathogenic Th1 and Th17 cells has been documented in MS and EAE (16, 39). Baricitinib administration potently diminished the polarization of Th17 cells and significantly inhibited the differentiation of Th1 cells in CNS and spleen (**Figures 3A, C**; **Figures 5A, C**). This inhibition alleviated the severity of EAE. T cells from EAE mice treated with baricitinib have a reduced encephalitogenic potential compared with T cells from EAE mice treated with vehicle. Our study showed that the decreased encephalitogenic potential of baricitinib coincided with reduced mRNA expression of RORγt and T-bet without increasing the mRNA expression of GATA and Foxp3 in the splenic JAK/STAT axis and may serve as a target for therapeutic intervention in MNCs (**Figures 5E–H**). RORγt is a critical transcription factor affecting Th17 cell polarization; the mRNA levels of RORγt decreased and the percentage of Th17 cells decreased. Baricitinib treatment potently suppressed Th17 cell subsets. T-bet plays a pivotal role in proinflammatory Th1 cell differentiation. Baricitinib can downregulate Th1 cell subsets, which may help alleviate the severity of EAE.

Improvement in the clinical symptom scores was associated with the inhibition of inflammatory responses in the spleen, indicating that suppression of the JAK/STAT pathway affects pathogenic T cells (**Figures 1A–D**). Baricitinib treatment inhibited the phosphorylation of JAK1, JAK2, STAT1, STAT3, and STAT4 without altering the total levels of JAKs and STAT proteins (**Figure 6B**). A reliable parameter of JAK inhibition is the downstream inhibition of STAT activation (40). STATs activation is essential for the differentiation of Th1 and Th17 cells (15, 41). STAT3 signaling plays a central role in Th17 cell differentiation. Th1 differentiation is activated by STAT1 and STAT4 (42). Baricitinib inhibits the JAK/STAT pathway and differentiation of Th1 and Th17 cells.

The pathogenesis of MS and EAE is involved in the overexpression of cytokines, including IL-12, IFN-γ, IL-6, IL-21, and IL-23, which promote the differentiation of effector Th1 and Th17 cells (43). Using ELISA, our present work showed that baricitinib exerted a significant effect on the cytokine profile in mice with EAE. Inflammatory cytokine production (IL-2, IL-12, IL-6, IL-17, IL-23, TNFα, and IFN-γ) was noticeably reduced in the therapeutic group compared to that in the control group (**Figure 7**). Baricitinib treatment inhibits Th1 cell differentiation, which is related to the inhibition of IFN-γ, IL-2, and IL-12 signaling. Th1 cell differentiation requires IL-12 through the activation of JAK2 and its downstream STAT4. Baricitinib is a potent inhibitor of IL-6 and IL-23 signaling, which are critical for Th17 cell polarization (44). Th17 differentiation is induced by IL-6 and/or IL-23 through JAK1/2 and STAT3 (45). These results suggest that the amelioration of the inflammatory environment by baricitinib is the basis for its protective effect against EAE. Our study showed a reduction in Th1-type cytokines and Th17 production without an increase in Th2-type cytokines and Tregs (**Figure 5**). This indicates that inhibition of the JAK/STAT pathway influences the immunological cascade in the early stages, leading to EAE. This inhibition leads to a reduction in immune cell infiltration into the CNS. Thus, inhibition of STAT activation may be responsible for decreasing inflammatory lesions and reducing proinflammatory mediators. Baricitinib treatment diminished T cell proliferative capacity, which contributed to its therapeutic effectiveness (**Figure 5I**). The immunomodulatory effect of baricitinib was associated with the reduced polarization of Th1 and Th17 cells, diminished expression of proinflammatory cytokines, and decreased infiltration of immune cells.

Baricitinib crosses the blood brain barrier and potently decreases human immunodeficiency virus-induced neuroinflammation with a decrease in activated microglia and astrocytes (GFAP) conferred on human immunodeficiency virus-infected mice (46). Baricitinib shows a rapid and remarkable suppression of the JAK2/STAT3 pathway in microglia *in vitro* (47). We evaluated the effect of baricitinib on the quantity of microglia and astrocytes in the spinal cord of EAE mice and found that baricitinib treatment potently reduced the quantity of microglia and astrocytes in CNS (**Figures 4A, B**). The protection from disease in baricitinib-treated mice might not depend only on its effect on T cells and may also affect the innate and adaptive immune system. In this study, we focus on the management of Th1- and Th17-mediated inflammation in EAE. Further study is necessary about the effect of baricitinib on other innate immune cells and the concomitant impairment of both adaptive and innate immune responses in the future.

Studies have shown that the JAK/STAT axis regulates the severity of EAE (48–50). Many JAK inhibitors have been investigated in EAE. Tofacitinib, a JAK inhibitor, has been approved by the Food and Drug Administration for the treatment of autoimmune diseases (51, 52). Baricitinib has also been approved in Europe and Japan for the treatment of adults with RA (53). Tofacitinib broadly interferes with Th1 and Th2 differentiation and impairs the production of Th17 cells (54). The protective effect of copolymer I in EAE partly inhibits STAT4 and STAT3 phosphorylation in T cells, suppressing Th1 and Th17 cell differentiation (55). Several herbal compounds and pyridinol derivatives, including plumbagin, berberine, and BJ-2266, exert protective effects in EAE disease models by inhibiting STAT activation and repressing Th1 and Th17 cell differentiation (56–58). In this study, we used a specific inhibitor of JAK1 and JAK2 in the JAK/STAT pathway. Baricitinib treatment was administered at the onset of the disease, with potent clinical efficacy. These findings collectively demonstrated baricitinib inhibiting the JAK/STAT axis in MS. Baricitinib could be an effective therapeutic approach for treating T cell-mediated autoimmune diseases.

Baricitinib, a JAK1 and JAK2 inhibitor, has some advantages over immune drugs for MS therapy. Baricitinib provides rapid improvements in RA symptoms and disease activity (59), and is generally well tolerated with long-term efficacy (60). Compared with subcutaneous injection of glatiramer acetate, baricitinib is an oral drug that is to be taken once daily and is convenient (22). Unlike numerous undesirable effects of finglimod, including bradycardia, atrioventricular block, and macular edema, baricitinib shows better safety (61, 62). Laquinimod, as a potential immunomodulator, failed to reach the primary endpoint of decrease in disability progression in a clinical trial of patients with relapsing MS (63, 64). The development of laquinimod will probably be challenging in MS. Baricitinib is a promising therapeutic drug for MS therapy.

In conclusion, we provide evidence that the immunomodulatory effect of baricitinib involves decreased polarization of Th1 and Th17 cells, diminished expression of proinflammatory cytokines, and decreased infiltration of inflammatory cells, thus decreasing the severity of EAE. Our findings show that baricitinib is a promising therapeutic target for MS and should be investigated further.

## Data Availability Statement

The raw data supporting the conclusions of this article will be made available by the authors, without undue reservation.

## Ethics Statement

All experiments were approved by the Animal Experiments Ethical Committee of Chengdu University of Traditional Chinese Medicine.

## Author Contributions

CD designed the research. QL analyzed the data. CD, YL and QL performed the research. CD wrote the paper. XC contributed new reagents and analytical tools. YL developed the software necessary to perform and record the experiments. All authors contributed to the article and approved the submitted version.

## Funding

This study was supported by Health Commission of Sichuan Province 20PJ195 and National Natural Science Foundation of China (82001240).

## Conflict of Interest

The authors declare that the research was conducted in the absence of any commercial or financial relationships that could be construed as a potential conflict of interest.
